# The Hormon Treatment of Post-Operative Intestinal Paralysis

**Published:** 1911-07

**Authors:** 


### The Hormon Treatment of Post-Operative Intestinal Paralysis

THE study of the hormons constitutes a fascinating chapter in modern physiological research. These bodies, as the name indicates, may be considered as nature's means of stimulating the functions of certain organs. Thus, for instance, it has been shown by Bayliss and Starling that the duodenal mucous membrane produces a "secretin" which when injected intravenously stimulates the pancreatic secretion, while adrenalin besides its various other actions also officiates as a hormon in the metabolism of the sugars. It would appear, therefore, that apart from their regular secretory activity many organs generate substances which are carried by way of the circulation to distant parts, and there officiate as functional stimulants.

One of the most interesting discoveries is that of Zuelzer, Dohrn and Marxer that the mucous membrane of the stomach contains a hormon, which when injected intravenously was found to stimulate intestinal peristalsis. It was later demonstrated that the same hormon can be extracted from the spleen in amounts sufficient to enable it to be utilised therapeutically. This discovery is of interest both to the physician and surgeon in the treatment of intestinal obstruction from atony of the bowel, as well as of post-operative intestinal paralysis. The results reported by Zuelzer and Saar with Hormonal, the name under which this hormon product has been introduced, have been most encouraging. Its advantage over physostigmin, which has been employed for the same purpose is that it produces a natural peristalsis and not one of tetanic character. While symptoms of reaction, such as fever and headache, have been noted from its use, these have been of slight and transient nature, and there is every reason to believe that this new physiologic product will prove of material service in medical and surgical practice.—(*International Journal of Surgery,* April, 1911.)

